# Analysis of Pepsin Concentration and Influencing Factors in Saliva of Elderly Nasal Feeding Patients

**DOI:** 10.1155/2021/4721812

**Published:** 2021-01-25

**Authors:** Yu Ding, Huiru Hou, Miao Liu, Xiaoyuan Wang, Yue Xu, Haiyan Shi, Haitao Du, Liyuan Wang

**Affiliations:** ^1^Department of Digestive, The Second Medical Centre, Chinese PLA General Hospital, Beijing, China; ^2^The Second Medical Centre, Chinese PLA General Hospital, Beijing, China; ^3^Institute of Geriatrics, Chinese PLA General Hospital, Beijing, China; ^4^Medical School of Chinese PLA, Beijing, China

## Abstract

**Background:**

Elderly patients receiving nasal feeding have weaker physiological function, and placement of a nasogastric tube weakens the natural barrier of the cardia-esophageal sphincter; therefore, the risk of gastroesophageal reflux (GER) is higher. Many studies have shown that pepsin is extremely sensitive in predicting GERD, so this study intends to investigate the level of pepsin in saliva of elderly patients with nasal feeding and analyze its influencing factors.

**Methods:**

This was a cross-sectional study. Patients admitted to the Chinese PLA General Hospital from April 2018 to October 2018 who received nasal feeding were included. One ml of saliva was collected from each patient in while sitting during fasting in the morning and 1 hour after lunch for 3 consecutive days. Pepsin was quantified by enzyme-linked immunosorbent assay (ELISA). The patients were predivided into two groups (≥7.75*μ*g/ml or <7.75*μ*g/ml) based on the median pepsin. Baseline and clinical factors were compared.

**Results:**

The mean age of the patients was 91.09 ± 4.91 years. There were statistical differences in diabetes and feeding methods between the two groups. There was a positive correlation between the morning and postprandial pepsin levels (*r* = 0.442, *P* < 0.001), and has no statistical difference (*P* = 0.175). Multivariate analysis showed that the risk factors for higher pepsin levels were diabetes (odds ratio (OR): 2.67; 95% CI: 1.225-5.819, *P* = 0.013) and nasal feeding methods (OR: 2.475; 95% CI: 1.183-5.180, *P*=0.016).

**Conclusions:**

For patients undergoing nasal feeding who are older than 80 years, the fasting and 1-hour postprandial pepsin concentration were consistent. Diabetes and feeding methods are risk factors for high pepsin levels. For the elderly over 80 years old, age has no influence on pepsin concentration.

## 1. Introduction

The elderly aged over 80 have the highest prevalence and fastest growing rates of disability [1]. Due to physiological dysfunction and multiple diseases, it is often necessary to support these patients with enteral nutrition. Nasogastric gavage (NG) is a common method of providing enteral nutrition; however, placement of the nasogastric tube can weaken the natural barrier of the cardia lower esophageal sphincter, increasing the risk of food and stomach acid reflux [2]. Moreover, elderly patients with chronic diseases have prominent comorbidities and severe physiological decline which increases the risk of gastroesophageal reflux disease (GERD) [3].

Diagnosis of GERD usually involves the assessment of a combination of clinical symptoms, response to acid suppression, and objective testing with upper endoscopy and esophageal pH monitoring [4]. However, invasive methods, such as esophageal reflux monitoring, endoscopy, and esophageal manometry, can be difficult, while noninvasive methods, including a GERD diagnostic questionnaire and proton pump inhibitors (PPI), may not be accurate. Thus, diagnosis can be inappropriate, expensive, and painful [5]. Elderly patients often have complicated conditions, and their symptoms are atypical, which can also increase the difficulty in diagnosis of GERD [6]. In recent years, a number of studies have confirmed that human salivary pepsin concentration can be used as a noninvasive, economic, fast, and effective method for diagnosing GERD [7–9]. Li et al. [10, 11] detected the pepsin concentration in human saliva by enzyme-linked immunosorbent assay (ELISA). The sensitivity of pepsin in the diagnosis of GERD was 93.8%, and the specificity was 46.2%. This suggested that the sensitivity of pepsin concentration was superior to a diagnostic questionnaire and proton pump inhibitor (PPI) test. Hayat et al. [5] reported that the postprandial pepsin level more accurately reflected GERD, which can be used as an auxiliary diagnostic method for GERD, thereby reducing the need for invasive and expensive diagnostic procedures.

Given the importance of pepsin, this study is aimed at investigating the level of pepsin in saliva of elderly patients with nasal feeding and analyze its influencing factors and is hoping to provide help for the clinical practice of elderly patients with nasal feeding.

## 2. Methods

### 2.1. Patients

This was a cross-sectional study. Patients who received enteral nutrition through a nasogastric tube from August 2018 to September 2018 at the Chinese PLA General Hospital (Beijing, China) were enrolled. The study protocol was approved by the Ethics Committee of the Chinese PLA General Hospital (S2018-097-01), and all patients or their families signed informed consent.

### 2.2. Inclusion and Exclusion Criteria

The following are the inclusion criteria: (1) aged ≥80 years; (2) nasal feeding through a nasogastric tube for more than 1 month; (3) stable condition during this study without fluctuations in vital signs; and (4) had not drink alcohol, smoked, or eaten spicy food during the past six months. The following are the exclusion criteria: (1) patients with gastrointestinal decompression, (2) patients with major gastrectomy, or (3) patients who underwent the PPI test in the past month.

### 2.3. Study Design

Patients were tested for pepsin concentration for 3 consecutive days, during fasting in the morning and 1 h after lunch while sitting (a total of 6 saliva samples were collected from each patient).

The mean values of pepsin concentrations during fasting in the morning and 1 h after lunch were calculated, respectively, for each patient. Patients were divided into 2 groups based on the median of the mean values of pepsin concentrations. Baseline and clinical factors were compared between the groups.

### 2.4. Data Collection

A unified data collection form was used to gather general clinical data from patients based on case data, nursing records, and bedside visits. Data collected included age, history of diabetes, history of hypertension, mechanical ventilation, total daily feeding volume, feeding methods, the ratio of the length of internal gastric tube to its height, type of gastric tube type, and duration of gastric tube placement.

### 2.5. Specimen Collection and Processing

Two trained nurses collected the saliva specimens. In order to avoid the effects of teeth brushing and mouthwash on the pepsin concentrations in the mouth, a suction tube with vacuum aspiration was used to suck out saliva from the throats of all patients. No specimen contained less than 1 ml. The specimens were stored at 4°C and mixed 4 times with 0.1% dithiothreitol (DTT) for 30 min. After using a 37°C water bath for 10 min, the mixture was centrifuged at 4°C and 5000 rpm for 7 min, and the supernatant was tested. The concentration of pepsin in the supernatant was detected by ELISA. The human secretion pepsin ELISA kit was purchased from Beijing Jing lai Hua ke Biological Co., Ltd. (Beijing, China). The automatic microplate reader was Denley Dragon Wellscan MK 3 (Thermo, Finland), the Thermo Scientific Wellwash 4 Mk2 microplate washer was used (Thermo, Finland), and the data were analyzed by Ascent software (Thermo LabSystems Inc., MA, USA).

### 2.6. Statistical Analysis

Continuous variables underwent a probability (PP) plot, quantile-quantile (Q-Q) plot, and the Shapiro-Wilk test for normality. Variables which conform to normal distributions were expressed as the mean ± standard deviation (SD) and compared with Student's *t*-test. Variables which do not conform to normal distributions were expressed as the median (lower quartile, upper quartile) and compared with Mann–Whitney *U* test. Categorical variables were expressed as the number and percentages and compared with a chi-square test. Spearman's correlation analysis was performed to investigate the association of fasting and postprandial pepsin levels in saliva, because they did not conform to normal distributions. A logistic regression model was used for univariate and multivariate analysis to find independent factors affecting higher pepsin levels. Statistical significance was defined at *P* < 0.05. All data were analyzed using SPSS 17.0 software (SPSS, Chicago, USA).

## 3. Results

### 3.1. Inclusion of the Participants

**Figure 1:**
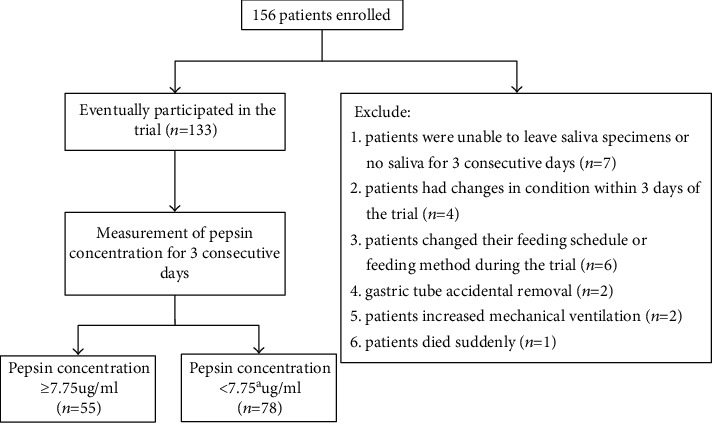
Flow chart showing the inclusion of patients in the study.

There were 156 male patients who met the inclusion criteria and were enrolled. During the study, 23 patients were excluded due to various reasons. Seven patients were unable to produce saliva specimens for 3 consecutive days, 4 patients had changes in condition during this study, 6 patients changed their feeding frequency or feeding method, 2 patients received accidental percutaneous endoscopy gastrostomy tube removal, 2 patients presented with increased mechanical ventilation, and 1 patient died suddenly (Figure 1).

### 3.2. Clinical Characteristics in the Two Groups

Table 1 shows the clinical characteristics of the 133 patients. As a positive threshold for salivary pepsin diagnosis of GERD has not been determined, we divided the patients into 2 groups, ≥7.75 *μ*g/ml or <7.75 *μ*g/ml, based on the median pepsin concentration. There were no statistical differences in age between the 2 groups (91.8 ± 4.37*vs.*90.8 ± 4.51, *P* = 0.220), whereas statistical differences were found in diabetes (*P* = 0.022) and feeding methods (*P* = 0.034) between the two groups.

### 3.3. Comparison of Fasting and Postprandial Pepsin Levels

The fasting and postprandial pepsin levels were 4.053 (2.163; 8.467)*μ*g/ml and 5.108 (2.458; 7.928)*μ*g/ml, respectively, showing no significant differences (*P* = 0.175). Spearman's correlation coefficient (*r*) was equal to 0.442 (*P* < 0.001), indicating a positive correlation between these two measurements.

### 3.4. Logistic Multivariate Regression Analysis of Pepsin Content in Elderly Patients with Long-Term Nasal Feeding

Taking the pepsin concentration as the dependent variable, variables with *P* < 0.1 in the univariate analysis were selected as the independent variables and included in the logistic regression model.

The results showed that the main factors influencing the pepsin concentration in saliva of elderly patients with long-term nasal feeding were diabetes (OR: 2.670, CI: 1.225-5.819) and nasal feeding method (OR: 2.475, CI: 1.183-5.180) (Table 2).

## 4. Discussion

This cross-sectional study involved 133 patients aged over 80 undergoing nasal feeding. We measured the pepsin level in saliva of these patients and investigate influencing factors. We found that factors independently related to a relatively high level of pepsin in saliva were diabetes (OR: 2.67; 95% CI: 1.225-5.819, *P* = 0.013) and nasal feeding methods (OR: 2.475; 95% CI: 1.183-5.180, *P* = 0.016). In addition, we also found that the salivary pepsin concentration of elderly patients with nasal feeding is higher than the reported cut-off value (0.1081 *μ*g/ml) [11]; this result also indirectly indicates that these people are at a higher risk of GERD.

Invasive tests for GERD diagnosis are difficult and even impossible in very elderly patients, such as those included here, so we used the pepsin level as an indicator of GERD. Due to a lack of a gold standard for a normal pepsin level in elderly people, the patients were divided into two groups according to the median pepsin level. Previous studies have suggested that the concentration of pepsin in saliva samples changes at different time periods [5, 12]; however, there is still no consensus on the optimal collection time [5, 8, 9]. Hayat et al. [5] used a PPI test to determine pepsin and suggested that the positive detection rate and concentration 1 h after a meal are higher than during the fasting state in the morning; therefore, they recommended collecting saliva samples 1 h after meal. However, Na et al. [12] believed that the concentration of pepsin upon waking was higher than 1 h after a meal when GERD symptoms occur. To resolve this difference, we collected the patient's samples during fasting in the morning and 1 h after lunch. The results showed that the pepsin levels in the morning and 1 h after lunch were highly positively correlated, with no significant difference (*z* = −1.355, *P* = 0.175). This difference with the previous studies might be because of the differences in the populations. In elderly patients with nasal feeding, the concentration of pepsin accumulated in the mouth and throat likely remained relatively stable due to decreased activity, increased time in bed, and decreased saliva secretion. Therefore, we speculate that the pepsin content in the saliva of elderly patients with nasal feeding is basically stable throughout the day and responds to reflux.

Age was not independently related to increased salivary pepsin in this study. However, other studies on different age groups have suggested that age does have an influence GERD. In a previous study, prevalence was significantly higher in subjects aged more than 50 years [13]. Another study [3] indicated that increased age was associated with the prevalence of GERD, and the mechanisms of increased GERD disease in older patients intensified the underlying diseases, disturbed esophageal motility, and decreased salivary secretion. However, there are few studies concerning patients aged ≥80 years. In our study, age was not a risk factor because there was no significant difference in the degree of underlying diseases and physiological functions of the patients aged ≥80 years. In other words, when patients are aged ≥80 years, their condition is complex, which attenuates or disperses the effects of age.

According to a previous study, diabetic patients are more prone to GERD [14]. This is in agreement with the results of this study which found that diabetes was related to higher levels of pepsin in the saliva. Hyperglycemia affects autonomic function and gastrointestinal hormone secretion, resulting in insufficient gastric motility in patients [3, 15, 16]. Insufficient gastric motility is one pathogenic mechanism of GERD. In addition, obesity is an important risk factor for GERD, and obesity and type 2 diabetes are closely associated [14]. This suggests that patients with a history of diabetes should be specifically concerned with the presence of GERD during medical and nursing care.

Feeding method was also shown to be related to a higher level of pepsin in saliva. It has been reported that the use of a stomach tube with a small outer diameter can reduce the occurrence of complications, such as reflux [17]. However, the results of this study showed that the outer diameter of the gastric tube was not a factor affecting the content of pepsin. Perhaps, the sample size of this study was too small, and the diameters of the two gastric tubes were not remarkably different. Therefore, we could not reach a similar conclusion. Previous studies in Chinese have suggested that a nasal feeding pump can inject liquid food into the stomach at a constant rate and slowly, which is superior to syringe injection in preventing intestinal nutrition complications. The results of our study also showed a high risk of high concentrations of pepsin in the saliva of syringe-fed patients. We hypothesize that because syringe feeding injects food into the stomach quickly, causing a rapid increase in the pressure applied to stomach, this leads to the increased risk of GERD. In contrast, using a nasal feeding pump not only reduces the pressure of the food on the lower esophageal sphincter but also slows down the rate of blood glucose in the patient.

If we detect high concentration of pepsin in the saliva, this means that the patient was prone to GERD or already suffers from GERD; we will recommend him to accept the gold standard diagnosis of GERD and, at the same time, avoid other risk factors and take corresponding measures, such as using a nasal feeding pump or other methods to EN, so as to avoid causing more serious complications, such as aspiration pneumonia.

There were several limitations in this study. First, the sample size was not very large, and we did not compare the results with a healthy control group. Second, due to the gold standard diagnostic method for GERD being invasive and not suitable for patients aged 80 and over, we could only choose the level of pepsin in saliva to indicate GERD. As the level of pepsin that indicates GERD has not been agreed, we cannot definitely say that the patients in the ≥7.75 *μ*g/ml pepsin group had GERD. Third, this was a cross-sectional study and lacked follow-up data. Fourth, the patients included in this study were all male, and the results might not apply to female patients. Finally, only the use of nonsteroidal anti-inflammatory drugs (NSAID), aspirin, and anticholinergic drugs on the level of pepsin was analyzed. Other drugs with potential effects were not included in the analysis. These may cause some bias to the results.

## 5. Conclusions

In conclusion, for patients undergoing nasal feeding who were older than 80 years, diabetes and feeding methods were the risk factors for the increased pepsin level; if such patients do not take it seriously, they may be prone to GERD. For nasal feeding patients over 80 years old, age has a weaker effect on pepsin concentration; at the same time, the concentration of pepsin in saliva remains the same throughout the day and has not changed due to feeding.

## Figures and Tables

**Table 1:** Demographic and clinical characteristics of the 133 enrolled patients.

Variables	Pepsin concentration ≥ 7.75 *μ*g/ml^a^ (*n* = 55)	Pepsin concentration < 7.75 *μ*g/ml (*n* = 78)	*P*
Age (year)	91.8 ± 4.4	90.8 ± 4.5	0.220
Esophageal hiatal hernia	2 (3.63)	2 (2.56)	0.721
Hyperlipidemia	7 (12.72)	11 (14.10)	0.819
Family history of gastroesophageal disease	4 (7.27)	7 (8.97)	0.726
OSAS	1 (1.82)	1 (1.28)	0.802
Drugs related to GERD^b^	16 (29.09)	29 (37.18)	0.332
Diabetes	14 (25.45)	35 (44.87)	0.022
Hypertension	38 (69.09)	48 (61.54)	0.370
Mechanical ventilation	19 (34.55)	36 (46.15)	0.213
Total daily feeding volume (ml)			0.297
≤500	6 (10.91)	16 (20.51)	
501-1500	35 (63.63)	47 (60.26)	
>1500	14 (25.45)	15 (19.23)	
Feeding method			0.028
Syringe injection	36 (65.45)	36 (46.15)	
Nasal pump feeding	19 (34.55)	42 (53.85)	
Insertion length/height			0.774
<0.35	24 (43.64)	36 (46.15)	
≥0.35	31 (56.36)	42 (53.85)	
Tube diameter (mm)			0.092
4.46	30 (4.55)	31 (39.74)	
3.23	25 (45.45)	47 (60.26)	
Stomach tube placement time (day)			0.062
≥512^c^	22 (0.4)	44 (56.41)	
<512	33 (0.6)	34 (43.59)	

OSAS: obstructive sleep apnea syndrome; GERD: gastroesophageal reflux disease. ^a^7.75 *μ*g/ml is the median of pepsin content. ^b^Mainly including nonsteroidal anti-inflammatory drugs, aspirin, and anticholinergic agents. ^c^512 days is the median time of stomach tube placement (day).

**Table 2:** Multivariate analysis of factors influencing pepsin in saliva.

Factors		OR	95% CI	*P*
Diabetes		2.670	1.225-5.819	0.013
Feeding methods	Nasal pump feeding	1		
	Syringe injection	2.475	1.183-5.180	0.016

OR = odds ratio; CI = confidence interval.

## Data Availability

The datasets used and/or analyzed during the current study are available from the corresponding author on reasonable request.

## Abbreviations

GERD: Gastroesophageal reflux disease
ELISA: Enzyme-linked immunosorbent assay
NG: Nasogastric gavage
PPI: Proton pump inhibitors
DTT: Dithiothreitol
PP: Probability plot
Q-Q: Quantile-quantile
SD: Standard deviation
NSAID: Nonsteroidal anti-inflammatory drugs.
